# Microvesicles Derived from Indoxyl Sulfate Treated Endothelial Cells Induce Endothelial Progenitor Cells Dysfunction

**DOI:** 10.3389/fphys.2017.00666

**Published:** 2017-09-08

**Authors:** Andres Carmona, Fatima Guerrero, Paula Buendia, Teresa Obrero, Pedro Aljama, Julia Carracedo

**Affiliations:** ^1^Maimonides Institute of Biomedical Research of Cordoba Cordoba, Spain; ^2^Department of Nephrology, Nephrology Service, Reina Sofia University Hospital Cordoba, Spain; ^3^RETICs Red Renal, Instituto de Salud Carlos III Madrid, Spain; ^4^Department of Animal Physiology II, Faculty of Biology, Complutense University of Madrid Madrid, Spain

**Keywords:** indoxyl sulfate, endothelial microvesicles, endothelial progenitor cells, endothelial dysfunction, miRNAs

## Abstract

Cardiovascular disease is a major cause of mortality in chronic kidney disease patients. Indoxyl sulfate (IS) is a typical protein-bound uremic toxin that cannot be effectively cleared by conventional dialysis. Increased IS is associated with the progression of chronic kidney disease and development of cardiovascular disease. After endothelial activation by IS, cells release endothelial microvesicles (EMV) that can induce endothelial dysfunction. We developed an *in vitro* model of endothelial damage mediated by IS to evaluate the functional effect of EMV on the endothelial repair process developed by endothelial progenitor cells (EPCs). EMV derived from IS-treated endothelial cells were isolated by ultracentrifugation and characterized for miRNAs content. The effects of EMV on healthy EPCs in culture were studied. We observed that IS activates endothelial cells and the generated microvesicles (IsEMV) can modulate the classic endothelial roles of progenitor cells as colony forming units and form new vessels *in vitro*. Moreover, 23 miRNAs were contained in IsEMV including four (miR-181a-5p, miR-4454, miR-150-5p, and hsa-let-7i-5p) that were upregulated in IsEMV compared with control endothelial microvesicles. Other authors have found that miR-181a-5p, miR-4454, and miR-150-5p are involved in promoting inflammation, apoptosis, and cellular senescence. Interestingly, we observed an increase in NFκB and p53, and a decrease in IκBα in EPCs treated with IsEMV. Our data suggest that IS is capable of inducing endothelial vesiculation with different membrane characteristics, miRNAs and other molecules, which makes maintaining of vascular homeostasis of EPCs not fully functional. These specific characteristics of EMV could be used as novel biomarkers for diagnosis and prognosis of vascular disease.

## Introduction

Patients with chronic kidney disease (CKD) have a high incidence of cardiovascular disease (CVD). In fact, the mortality from CVD is very high in CKD patients even from the early stages of the disease when the patient does not present with symptoms (Foley et al., 1998; Wanner et al., 2016). Many uremic toxins are metabolites that bind to proteins (Ellis et al., 2016); in particular, indoxyl sulfate (IS), a protein-bound uremic toxin, is very difficult to remove by hemodialysis. Increased IS has been associated with the progression of CKD and the development of CVD (Niwa et al., 1997; Adijiang et al., 2008).

High levels of IS affect endothelial cells (EC), increasing the expression of adhesion molecules (Tumur et al., 2010) and oxidative stress that leads to endothelial damage *in vitro* (Tumur and Niwa, 2009) and *in vivo* (Yu et al., 2011). In fact, endothelial damage is considered a determinant stage for the development of CVD (Yu et al., 2011). Thus, the detection of morphological or functional alterations of EC is essential for the early diagnosis and prophylactic intervention of vascular complications in patients with CKD. However, it is difficult to check the endothelium status because of its inaccessibility. In the last few years, it was shown that EC release microvesicles (EMV), with characteristics that reflect the state of the cell they originated from (Faure et al., 2006; Gaceb et al., 2014). EMV are a subtype of extracellular vesicles produced by EC whose essential role is to serve as a signaling system for the function and homeostasis of the vessel (Meziani et al., 2008). EMV are involved in physiological and pathological processes on target cells by binding to ligands, surface receptors, and/or membrane associated enzymes, releasing their contents directly into the cytoplasm (Colombo et al., 2014). To maintain vascular homeostasis, damaged EC are replaced by endothelial progenitors cells (EPCs), which circulate in a low percentage in peripheral blood (Urbich and Dimmeler, 2004). This repair mechanism requires an exquisitely regulated intracellular signaling network that maintains an efficient balance between endothelial damage and the release of EPCs. Previous studies showed, in both animal and human endothelial injury models (Ramirez et al., 2007; Nogueras et al., 2008; Noci et al., 2015), a correlation between plasma levels of EMV and the activity of EPCs. We showed the development of severe vascular disease is associated with an increase in EMV that parallels the decrease in EPCs in patients with CKD (Soriano et al., 2014). Nevertheless, the factors involved are not known, and it is necessary to identify if uremic toxins, such as IS, could be involved in endothelial damage, releasing EMV that modulate endothelium repair.

Microvesicles (MV) can transfer proteins, cytokines, mRNAs, or miRNAs to target cells and influence their biological behavior (Hulsmans and Holvoet, 2013). miRNAs are highly conserved non-coding RNA molecules approximately 22 nucleotides long that exert post-transcriptional effects on gene expression. Importantly, MV represent major transport vehicles for miRNAs and their effects depend on the expression of the MV they are contained in (Cantaluppi et al., 2012; Diehl et al., 2012). miRNAs are highly expressed in EC, and recent data suggest that they regulate important aspects of vascular function. The objective of this study was to analyze, in an *in vitro* model, the effect of EMV derived from IS-treated human umbilical vein endothelial cells (HUVECs) on the endothelial repair process developed by EPCs.

## Materials and methods

### Human umbilical vein endothelial cells culture

Human umbilical vein endothelial cells (HUVECs) were obtained from Cell Systems (Clonetics, Solingen, Germany) and cultured at 37°C in a 5% CO_2_ atmosphere in EC basal medium (EBM) plus endothelial cell-growth medium supplements (EGM, Cambrex Bioscience, Walkersville, MD) and 10% fetal bovine serum (Invitrogen, Carlsbad, CA, USA). HUVECs were used for experiments between passages four and nine. HUVECs at 80% confluence were incubated with or without indoxyl sulfate (IS) at 256 μg/ml for 24 h. After the incubation period, cells were characterized for flow cytometry and culture supernatants were used for isolation of EMV. We first established the experimental model using a concentration- and time-response curve.

### Expression of endothelial adhesion molecules

Expression of ICAM-1, PECAM-1, VE-cadherin, and VCAM-1 were measured in HUVECs after 24 h of treatment with IS (256 μg/ml). Next, VE-cadherin (FITC rabbit anti human CD144, AbD Serotec, UK), PECAM-1 (PE-labeled monoclonal anti-CD31, Caltag Laboratories, Burlingame, CA, USA), ICAM-1 (PE mouse anti human CD54, Invitrogen), and VCAM-1 (PE Mouse Anti-Human CD106, BD Pharmingen) antibodies were used to assess the expression in the experimental conditions. HUVECs were incubated with the antibodies for 20 min in darkness at room temperature. Then, cells were washed with PBS and fixed with BD CellFIX (BD). HUVECs without antibody were used as a reference (negative control). Mean fluorescence intensity (MFI) of different antibodies was used to analyze the cytometer data.

### Endothelial microvesicles isolation

EMV from the culture medium of IS-treated and untreated HUVECs were isolated by ultracentrifugation. The media was centrifuged (Heraeus Labofuge 400R) at 409 g for 5 min at 4°C to remove any intact cells, followed by centrifugation at 789 g for 10 min at 4°C to remove cell debris. The media was then transferred to ultracentrifuge 25 × 89 mm polypropylene tubes (Beckman Coulter, Brea, CA, USA) and centrifuged at 18,000 g for 90 min at 10°C in an Optima XPN-100 ultracentrifuge with 70Ti rotor (Beckman Coulter). The EMV were sedimented owing to relative centrifugal forces. The supernatant containing EMV-free media was removed and the pellets containing EMV were resuspended in PBS and quantified by flow cytometry (FC500 Series, Beckman Coulter). Absolute values of MV were calculated using the following formula: (MV counted x standard beads/ L)/ standard beads counted (FlowCount, Beckman Coulter). Results were expressed as the number of MV per microliter of culture medium. MV derived from IS-treated HUVECs were defined as indoxyl sulfate EMV (IsEMV).

### Endothelial microvesicles analysis by flow cytometry

After 24 h incubation with or without IS (256 μg/ml), culture supernatants were collected. EMV were isolated as previously described. The pellet was resuspended in PBS and 10 μl aliquots were incubated with annexin V, ICAM-1, PECAM-1, VE-cadherin, or VCAM-1 for 20 min with gentle regular shaking at room temperature. EMV were quantified by flow cytometry (FC 500 Series). Prior to the sample acquisition, the samples were subjected to a separate and combined labeling reaction using all reactive (monoclonal antibodies, Annexin V, and the appropriate negative controls) to compensate for the fluorescence using compensation tools on the flow cytometer. In a previous study, we established a MV gate on the FC 500 cytometer using a blend of size-calibrated beads with diameters of 0.3, 0.5, and 1.0 μm (Carmona et al., 2017). The upper and outer limits of the MV gate were established just above the size distribution of the 1-μm beads in the forward (FSC-A) and side scatter (SSC-A) settings (log scale). The lower limit was the noise threshold of the instrument (SSC-A), limiting high background noise. The absolute number of MV was calculated as: (MV counted x standard beads/L)/standard beads counted (FlowCount, Beckman Coulter). Each result (single value) was the average of five independent measurements of the same sample.

### Isolation of miRNAs and the nanostring nCounter assay

miRNAs from EMV were extracted using the ISOLATE II miRNA kit Phenol free (Bioline, London, UK) according to the manufacturer's protocol. The NanoString nCounter® platform was used to screen the expression levels of 800 miRNAs using pools with miRNAs purified from controls and IsEMV. A total of 100 ng miRNAs (or 3 μl) was used per sample and conditions were set according to the manufacturer's recommended protocol (NanoString Technologies; Seattle, WA, USA). The nSolver 2.6 software was used to analyze and normalize the raw data using the top 100 most abundant miRNAs in all samples.

### Endothelial progenitor cells culture

All procedures performed were in accordance with the ethical standards of the institutional research committee and conformed to the standards set by the latest revision of the *Declaration of Helsinki*. Informed consent was obtained from all individual participants included in the study. EPCs were obtained from human peripheral blood mononuclear cells from healthy donors by density gradient centrifugation (Lymphoprep, Axis-Shield PoC, Oslo, Norway) and grown in EC basal medium-2 supplemented with growth factors (EGM-2 Bullekit, Lonza, Allendale, NJ, USA) and 15% autologous plasma. Mononuclear cells were plated onto fibronectin (BD, Franklin Lakes, NJ, USA) in coated, six-well plates at a density of 5 × 10^6^ cells/well and then incubated at 37°C in a 5% CO_2_ atmosphere for 3 weeks. Four days later, cells in suspension were removed and fraction of attached cells was cultivated with EBM-2 supplemented with 15% autologous plasma. On 7th day of EPCs culture, 10^4^ EMV or IsEMV (MV/ml) were added (Mezentsev et al., 2005). The medium and the two different stimuli were renewed every 2 days for 14 days. EPCs phenotype CD34+CD133+VEGFR2+ was verified with a cellular purity of >90%.

### Detection of hydroethidine

Hydroethidine (HE) (Invitrogen) is oxidized by ROS to become ethidium, which emits red color, and was used to measure superoxide anion. HUVECs and EPCs were exposed to HE (2 μM) for 15 min at 37°C. Quantitative analysis was performed on a flow cytometer (FACSCalibur, BD).

### Apoptosis quantification

The percentage apoptosis was measured by annexin V staining. HUVECs and EPCs were obtained by mechanical disruption and washed once with annexin V binding buffer (FITC Annexin V Apoptosis Detection Kit I, BD). Cells were then resuspended in annexin V binding buffer and annexin V was added following the manufacturer's protocol. Negative tube controls contained annexin V binding buffer. Quantitative analysis was performed on a flow cytometer (FACSCalibur, BD).

### Angiogenesis assay in matrigel

To evaluate the effect of MV on EPCs, *in vitro* angiogenesis was evaluated using the Endothelial Tube Formation Assay Kit (Cell Biolabs, San Diego, CA, USA). After of treatment with EMV or IsEMV, EPCs were obtained by mechanical disruption and seeded on semi-natural Matrigel. Briefly, 50 μl of thawed gel solution was added to each well of a pre-chilled 96 well sterile plate. For the angiogenesis assay, a total of 2,500 cells/well were plated, of which 50% were EPCs obtained from MV treated cultures, and the remaining 50% were mature endothelial cells at low passage stages (3–4). Vascular endothelial growth factor (50 ng/ml) was administered in parallel as an internal positive control. After 4 h, photographs were taken with an optical inverted microscope (OPTIKA Microscopes, Ponteranica, BG, Italy), and an automated analysis was performed with the ImageJ software (http://rsb.info.nih.gov/ij/). Four parameters were taken for the quantification experiments (Total length, NB segments, Nb branches and Nb master junctions). Results were expressed as previously described by Izuta et al. (2009).

### EPCs proliferation activity

After of treatment with EMV or IsEMV, the number of EPCs colony forming units (CFUs), characterized by a cluster of cells surrounded by elongated spindled-shaped cells, were counted manually by visual inspection using an optical inverted microscope (OPTIKA Microscopes, Italy) in a minimum of 10 random high-power fields.

### Protein expression analysis by western-blot

Cellular extracts from EPCs were prepared according to standard protocols (Andrews and Faller, 1991). Protein concentration was determined by the Bradford method (Bio-Rad Laboratories, Hercules, CA, USA). Cytoplasmic extracts (50 μg) or nuclear extracts (50 μg) were separated in a 4–20% Ready Gel Tris-HCl gel (Bio-Rad Laboratories), transferred onto nitrocellulose membranes in a semi-dry transfer system. The membrane was immediately placed into blocking buffer containing 5% nonfat milk and sequentially blotted against monoclonal primary antibodies (NFκB p65, IκBα, p53, and β-actin). All antibodies were from Cell Signaling (Boston, MA, USA) except β-actin (Santa Cruz, Dallas, TX, USA). Protein levels were quantified using the image analysis software Quantity One 4.4.0 (Bio-Rad Laboratories), using β-actin as a loading control.

### Statistical analysis

Data represent the mean ± SEM, and the analysis of variance (ANOVA) with a Bonferroni *post-hoc* correction was applied. Comparisons between paired or unpaired data were made by Student's-test. If the normality or equal variance test was violated, a comparison was made using the non-parametric Mann-Whitney *U*-test. *P*-values of < 0.05 were considered statistically significant. Statistical analyses were performed with SPSS 18.0 (IBM, Armonk, NY, USA).

## Results

### IS activates EC

**Figure 1:**
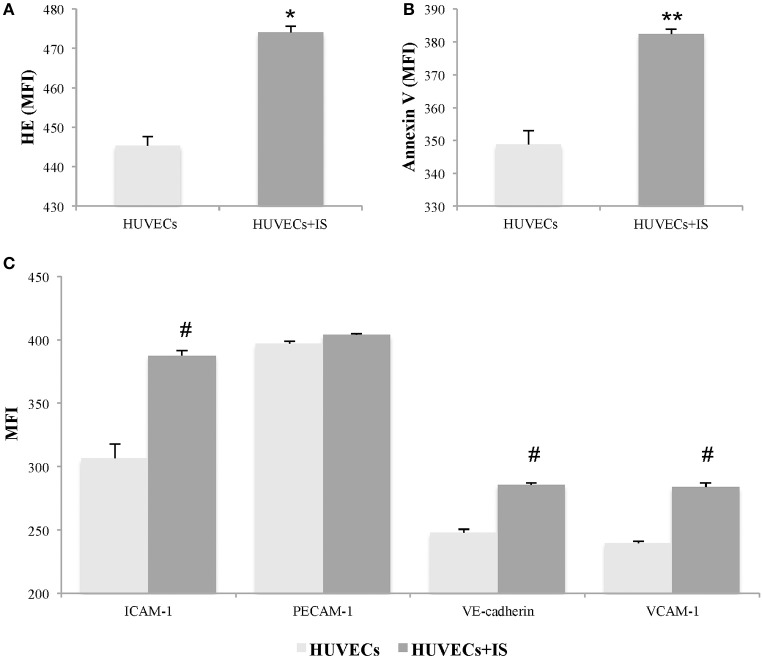
Indoxyl sulfate (IS) induces activation in human umbilical vein endothelial cells (HUVECs). HUVECs treated with IS (256 μg/ml) for 24 h showed significant increases in markers of oxidative stress and apoptosis. The expression was quantified by changes in the mean fluorescent intensity (MFI) of **(A)** hydroethidine (HE) and **(B)** annexin V. In addition, **(C)** IS induced significantly elevated expression of ICAM-1, VE-cadherin, and VCAM-1, but not PECAM-1. Data are the means ± SEM of five independent experiments. ^*^p = 0.004 vs. untreated HUVECs; ^**^p = 0.045 vs. untreated HUVECs; and ^#^*p* ≤ 0.001 vs. untreated HUVECs.

We evaluated whether IS mediates oxidative stress in EC, analyzing its effect on reactive oxygen species (ROS) production by flow cytometry. After 24 h of treatment, we observed a significant increase in the MFI of HE in IS-treated HUVECs compared with controls (474.0 ± 1.6 vs. 445.3 ± 2.3; *p* = 0.004) (Figure 1A). Furthermore, we quantified the binding of EC with annexin V and observed a significant increase in IS-treated HUVECs compared with controls (382.3 ± 1.4 vs. 348.6 ± 4.3; *p* = 0.045) (Figure 1B).

Moreover, we quantified the expression of adhesion molecules, indicative of endothelial activation, such as VCAM-1, ICAM-1, PECAM-1, and VE-cadherin. As shown in Figure 1C, IS induced increases in ICAM-1 (*p* < 0.001), VCAM-1 (*p* < 0.001), and VE-cadherin (*p* = 0.001) in HUVECs at 24 h. However, at 24 h of treatment with IS at 256 μg/ml, no changes were observed in the MFI of PECAM-1 compared with controls.

### Characterization of EMV release by IS

**Figure 2:**
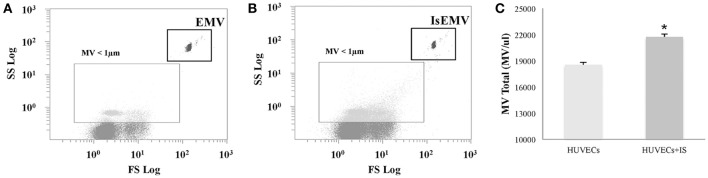
Human Umbilical Vein Endothelial Cells (HUVECs)-derived microvesicles (MV) assessed by flow cytometry. **(A)** Representative dot plot showing log forward scatter (FSC) vs. log side scatter (SSC) localization of MV. The upper right gate shows the bead flow count, used as an index to count MV in absolute terms. The lower left gate shows MV smaller than 1 μm. **(B)** Representative dot plot showing localization of MV derived from IS-treated HUVECs. **(C)** Absolute number of MV per microliter in IS-treated and untreated HUVECs. Results are the mean ± SEM of five independent experiments. ^*^p < 0.001 vs. MV derived from untreated HUVECs (EMV).

**Figure 3:**
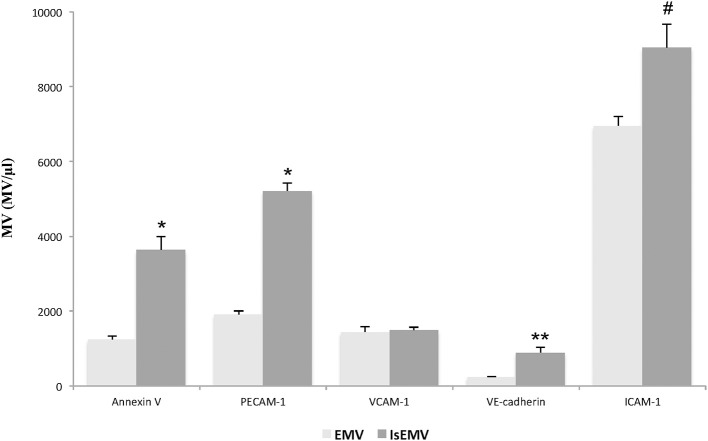
Indoxyl sulfate (IS) modulates the expression of adhesion molecules and annexin V in microvesicles (MV). MV (MV/μl) derived from indoxyl sulfate-treated HUVECs (IsEMV) showed a significant increase in annexin V and adhesion molecules, such as PECAM-1, VE-cadherin, and ICAM-1, but not VCAM-1. Results are the mean ± SEM of five independent experiments. ^*^p < 0.001 vs. MV derived from untreated HUVECs (EMV); ^**^p = 0.002 vs. EMV and ^#^*p* = 0.014 vs. EMV.

EMV release was related to endothelial activation. We observed elevated EMV release in IS-treated HUVECs (EMV/μl) compared with controls (21,741 ± 318 vs. 18,552 ± 285; *p* < 0.001) (Figures 2A–C). IS generated microvesicles (IsEMV) presented “endothelial-specific epitopes” such as ICAM-1, VCAM-1, PECAM-1, VE-cadherin, and annexin V. As shown in Figure 3, IsEMV induced increased levels of ICAM-1 (*p* = 0.014), PECAM-1 (*p* < 0.001), VE-cadherin (*p* = 0.002), and annexin V (*p* < 0.001). Although, VCAM-1 expression did not increase in IsEMV.

### Hierarchical cluster analysis of miRNAs

**Figure 4:**
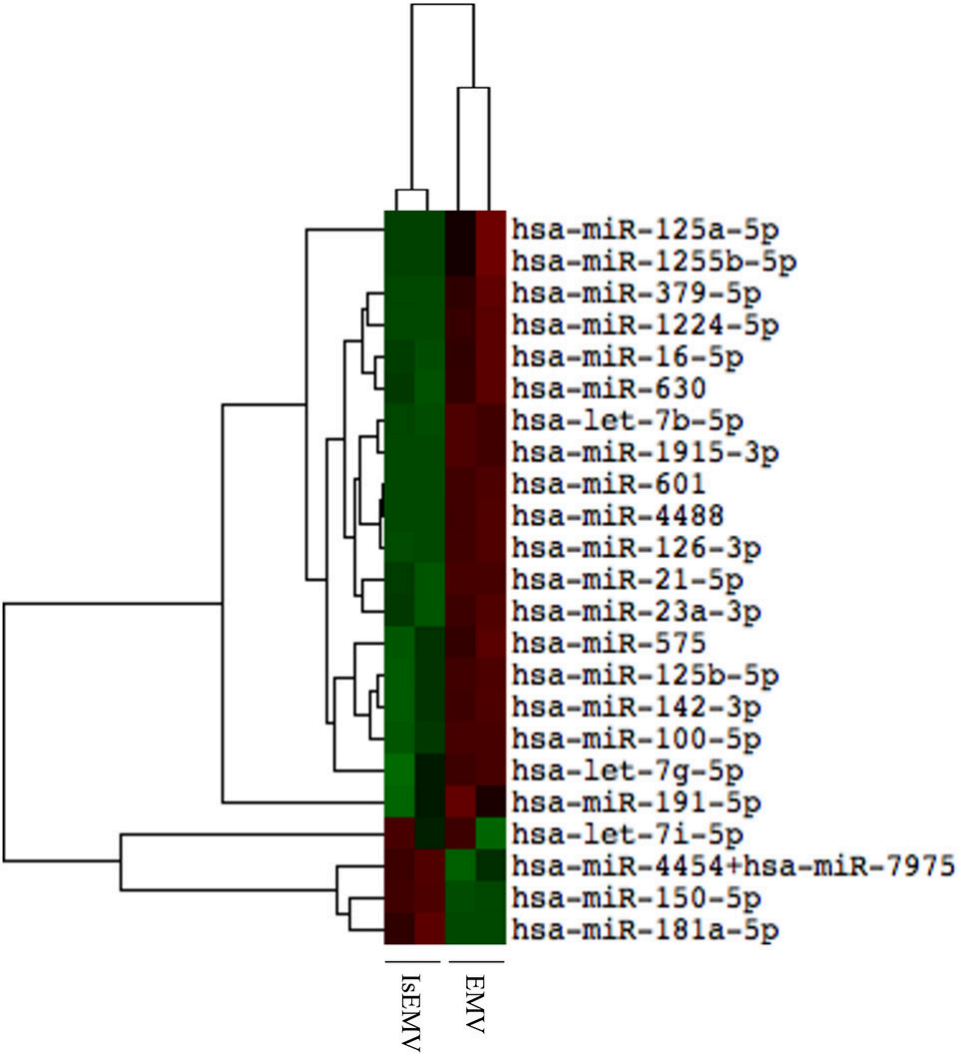
Hierarchical cluster analysis of miRNAs in microvesicles (MV). Heat map of differentially expressed miRNAs in MV derived from IS-treated HUVECs (IsEMV) and from untreated HUVECs (EMV). Each sample (*n* = 2) constitutes pooled cultured HUVECs. Red indicates upregulated miRNA expression and green indicates downregulated miRNA expression.

The nCounter profiling identified 23 miRNAs with differential expression in IsEMV relative to EMV (Table 1). Specifically, four overexpressed miRNAs (hsa-miR-4454+7975, hsa-miR-150-5p, hsa-miR-181a-5p, and hsa-let-7i-5p) and 19 downregulated miRNAs (hsa-miR-125a-5p, hsa-miR-1255b-5p, hsa-miR-379-5p, hsa-miR-1224-5p, hsa-miR-16-5p, hsa-miR-630, hsa-let-7b-5p, hsa-miR-1915-3p, hsa-miR-601, hsa-miR-4488, hsa-miR-126-3p, hsa-miR-21-5p, hsa-miR-23a-3p, hsa-miR-575, hsa-miR-125b-5p, hsa-miR-142-3p, hsa-miR-100-5p, hsa-let-7g-5p, and hsa-miR-191-5p) were identified in IsEMV. Hierarchic clustering was performed based on the 23 differentially expressed miRNAs and displayed as a heat map (Figure 4).

**Table 1:** Differential expression of screened miRNAs in microvesicles.

**Gene name**	**Fold change (IsEMV vs. EMV)**	***p*-value**	**Regulation**
hsa-miR-125a-5p	−1.17	0.21	Down
hsa-miR-1255b-5p	−1.17	0.21	Down
hsa-miR-379-5p	−1.51	0.11	Down
hsa-miR-1224-5p	−14.58	0.07	Down
hsa-miR-16-5p	−2.19	0.06	Down
hsa-miR-630	−1.61	0.03	Down
hsa-let-7b-5p	−2.15	0.002	Down
hsa-miR-1915-3p	−59.54	0.02	Down
hsa-miR-601	−43.98	0.03	Down
hsa-miR-4488	−27.15	0.03	Down
hsa-miR-126-3p	−2.81	0.02	Down
hsa-miR-21-5p	−7.51	0.05	Down
hsa-miR-23a-3p	−1.16	0.02	Down
hsa-miR-575	−15.07	0.03	Down
hsa-miR-125b-5p	−4.53	0.07	Down
hsa-miR-142-3p	−1.29	0.06	Down
hsa-miR-100-5p	−3.29	0.06	Down
hsa-let-7g-5p	−2.09	0.18	Down
hsa-miR-191-5p	−1.23	0.13	Down
hsa-let-7i-5p	1.16	0.71	Up
hsa-miR-4454+7975	2.39	0.07	Up
hsa-miR-150-5p	1.81	0.008	Up
hsa-miR-181a-5p	1.17	0.10	Up

*Four miRNAs were overexpressed and 19 downregulated in microvesicles derived from IS-treated HUVECs (IsEMV) compared with microvesicles derived from untreated HUVECs (EMV). Each sample (n = 2) constitutes pooled HUVECs cultures. miRNAs showing a fold change of at least 1.5 between IsEMV and EMV are represented. p < 0.05 indicates significant differences vs. EMV*.

### Effects of EMV on EPCs

**Figure 5:**
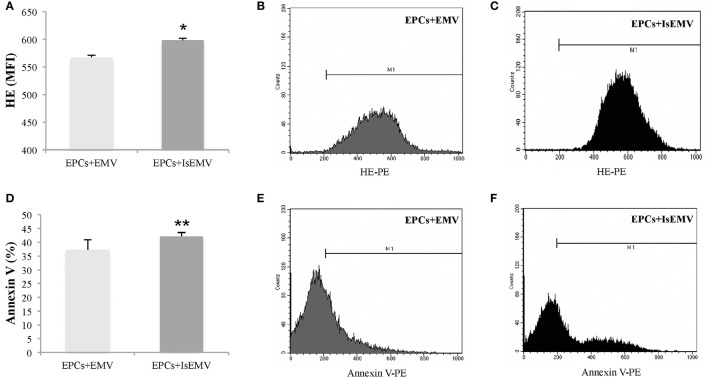
Effect of microvesicles (MV) on endothelial progenitor cells (EPCs). EPCs obtained from healthy donors were treated with MV derived from IS-treated HUVECs (IsEMV) or untreated HUVECs (EMV) for 14 days. The treatment with IsEMV increased markers of oxidative stress and apoptosis. **(A)** Hydroethidine (HE) expression analyzed by flow cytometry of EPCs treated with EMV or IsEMV. The expression was quantified by changes in the mean fluorescent intensity (MFI). Representative images of flow cytometry of HE expression in EPCs treated with **(B)** EMV and **(C)** IsEMV, M1 bars indicate cells positive for HE expression. **(D)** Differences in the percentage of annexin V in EPCs observed by flow cytometry in cultures treated with EMV or IsEMV. Representative images of flow cytometry of annexin V in EPCs treated with **(E)** EMV and **(F)** IsEMV, M1 bars indicate cells positive for annexin V expression. Results are the mean ± SEM of three independent experiments. ^*^p < 0.004 vs. EPCs treated with EMV; ^**^p = 0.045 vs. EPCs treated with EMV.

After 14 days of treatment with EMV or IsEMV, oxidative stress and apoptosis were quantified in EPCs. As shown in Figures 5A–C, we observed a significant increase in the MFI for HE in IsEMV-treated EPCs compared with controls (567.3 ± 3.7 vs. 598.3 ± 3.7; *p* = 0.004). Likewise, IsEMV induced a significant increased in the percentage of annexin V+ EPCs (37.3 ± 3.7 vs. 42.1 ± 1.4; *p* = 0.045) (Figures 5D–F).

### IsEMV modulate the *in vitro* progression of EPCs cultures

**Figure 6:**
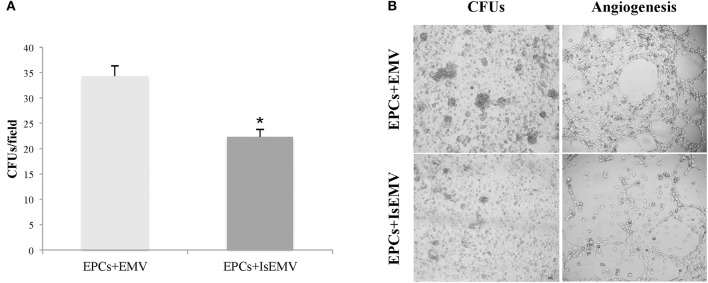
Effect of microvesicles (MV) on endothelial progenitor cells (EPCs) colony formation and angiogenesis. EPCs obtained from healthy donors were treated with MV derived from IS-treated HUVECs (IsEMV) or untreated HUVECs (EMV) for 14 days. **(A)** Histogram of colony forming units (CFUs) per field under different experimental conditions. IsEMV significantly decreased the number of CFUs. Results are the mean ± SEM of three independent experiments. ^*^p < 0.001 vs. EPCs treated with EMV. **(B)** Optical inverted microscopy images of CFUs in EPCs treated with EMV or IsEMV and representative images of tube-like three-dimensional structures of EPCs on the semi-natural matrix, Matrigel, 4 h after seeding.

We observed that IsEMV are able to modulate the differentiation of EPCs *in vitro*. Mononuclear cells from healthy donors were seeded onto fibronectin-coated plates. After 7 days in culture, the cells in suspension were removed and the fraction of attached cells was treated with EMV or IsEMV at 10^4^ MV/μl for 14 days. As shown in Figures 6A,B the treatment with IsEMV significantly decreased the number of colony forming units (CFUs) in EPCs compared with controls (34.3 ± 2 vs. 22.3 ± 2.1 CFUs/field; *p* < 0.001).

### Anti-angiogenic effect of IsEMV on EPCs

The ability of EMV to induce angiogenesis in EPCs was determined. As shown in Figure 6B, we observed that EPCs treated with IsEMV have a loss of angiogenesis; thus, EPCs developed a significantly lower number of vascular vessels in the 3D matrix. Values of different angiogenic parameters are shown in Table 2. Vascular endothelial growth factor (50 ng/ml) was used as an internal positive control (Data Supplementary Figure 1).

**Table 2:** Angiogenic parameters.

	**Total length**	**Nb segments**	**Nb branches**	**Nb master junction**
EPCs + EMV	15, 607.5 ± 1, 672.7	252 ± 43.6	66.7 ± 3.6	74.2 ± 10.7
EPCs + IsEMV	9, 541 ± 541	101 ± 7.2	50 ± 4.1	26.5 ± 2.9
*p*-value	0.007	0.027	0.012	0.002

*Tube formation was evaluated by measurement of total length, Nb segments, Nb branches, and Nb master junctions after treatment with microvesicles derived from untreated HUVECs (EMV) or microvesicles derived from IS-treated HUVECs (IsEMV). All analyzed angiogenic parameters had significant changes (p < 0.05) vs. EPCs treated with EMV*.

### IsEMV modulated p53, NFκB, and Iκbα in EPCs

**Figure 7:**
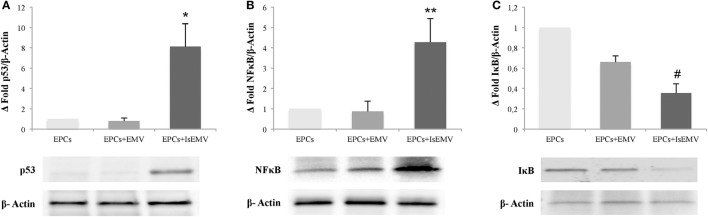
Microvesicles (MV) modulated p53, NFκB, and IκBα in endothelial progenitor cells (EPCs). Representative western blots and densitometry analysis of blots for **(A)** p53, **(B)** NFκB, and **(C)** IκBα. β-Actin was used as an internal control. IsEMV induced a significant increase in p53 and NFκB expression and significant decrease in IκBα expression in EPCs. Data are the means ± SEM of three independent experiments. ^*^p = 0.033 vs. EPCs treated with MV derived from untreated HUVECs (EMV); ^**^p = 0.022 vs. EPCs treated with EMV; and ^#^*p* = 0.044 vs. EPCs treated with EMV.

Immunoblot analysis of the expression of p53 protein, which regulates the cell cycle and apoptosis, is shown in Figure 7A. Protein expression of p53 in IsEMV-treated EPCs was significantly increased in comparison with the control group (*p* = 0.033). Control and EMV-treated EPCs showed normal expression of this protein. We also examined whether IsEMV treatment activated the NFκB pathway. Activation of the NFκB pathway is usually marked by the phosphorylation and degradation of the NFκB inhibitory protein, IκBα, releasing NFκB and allowing its migration to the nucleus (Hinz and Scheidereit, 2014). We observed that after IsEMV treatment, IκBα levels decreased in the cytosolic fraction (*p* = 0.044) and NFκB levels increased in nuclear fraction (*p* = 0.022) when compared with EMV-treated EPCs (Figures 7B,C).

## Discussion

Indoxyl sulfate is a uremic toxin associated with CVD during the progression of CKD (Niwa, 2013; Dou et al., 2015). In our study, we have established a model of endothelial damage using doses of IS to induce an increase in EMV release (Data Supplementary Figure 2). We demonstrated that EMV are involved in altering the repair process of endothelium in patients with CKD. In culture, HUVECs can be activated by IS, serving as an effective model to study the mechanisms involved in the pathophysiology of endothelial damage associated with CKD. Different studies have shown that EC have increased oxidative stress in response to progressive concentrations of IS (Vanholder et al., 2003; Dou et al., 2004; Peng et al., 2012; Lee et al., 2015). In agreement with previous studies, we found, in response to IS, EC have increased release of ROS after 24 h in culture. Other authors observed, at concentrations of IS similar to those found in CKD, an increase in ROS at shorter incubation times than we used (Dou et al., 2007; Tumur and Niwa, 2009). The increase in oxidative stress induced by IS and the associated release of ROS are related to activation of the EC. We observed an increase in membrane proteins, such as VCAM-1, ICAM-1, and VE-cadherin, as a result of activation and cell adhesion. Similarly, others have described a modulation of adhesion molecules on EC by IS in CKD (Lee et al., 2012) and CVD (Tumur et al., 2010). Further, we found an increase in apoptosis in IS-treated HUVECs, indicating that IS has a direct deleterious effect on EC. These results are consistent with prior studies in HUVECs (Tumur and Niwa, 2009; Lee et al., 2012), and mesangial cells (Wang et al., 2014).

In response to endothelial activation by IS, cells are capable of releasing a higher number of EMV into the medium. Increased levels of circulating EMV have been observed in pathologies associated with endothelial dysfunction, such as antiphospholipid syndrome (Dignat-George et al., 2004) and CVD (Mallat et al., 2000; Boulanger et al., 2006). This is also reported in patients with CKD and hemodialysis (Faure et al., 2006), suggesting that an excessive endothelial vesiculation may be indicative of endothelial dysfunction in uremia. Moreover, there is evidence that uremic toxins are capable of producing vesiculation of EC in culture and may induce endothelial dysfunction *in vitro* (Faure et al., 2006; Meijers et al., 2009; Gao et al., 2015). We characterized IsEMV and found their membranes, not only share molecules with the cells they originated from, but their expression can be modulated in different ways in response to activation. In this regard, we observed an increase in markers of endothelial adhesion and annexin V-binding in both IS-treated HUVECs and IsEMV. However, VCAM-1 increased in EC in response to IS, but not to IsEMV. On the contrary, IsEMV had a greater increase in expression of PECAM-1 than IS-treated HUVECs. We believe that this differential expression of molecules in the membranes of EMV is not random, and may be associated with their functionality.

It is known that MV plays an important role as messengers for signaling and cell communication (Burger et al., 2013; Erdbrügger and Le, 2016). Therefore, they may carry activation and apoptosis signals from HUVECs and exert specific effects on them or on other cells (Schock et al., 2014). A function that has been ascribed to EMV is the ability to recruit and differentiate EPCs (Chironi et al., 2010). EPCs are bone marrow-derived precursors placed as crucial mediators of the endothelial repair. In previous studies, we established the EPCs phenotype as events that were triple positive for CD133, CD34 and VEGFR2 (Soriano et al., 2014; Luna et al., 2015). Cells expressing these three molecules are the most commonly reported as “classical” EPCs (Friedrich et al., 2006; Zampetaki et al., 2008), but the differential expression of these molecules has been linked to different states of cellular differentiation as well as to the intensity of their repair capacity (Lanuti et al., 2016; Medina et al., 2017). Several studies reported that EPCs amount and functionality were decreased in patients with CKD reflecting a reduced capacity to repair the endothelium (Hill et al., 2003; Choi et al., 2004; Schmidt-Lucke et al., 2005; Soriano et al., 2014). Our study determined the functional changes on healthy EPCs in culture treated with EMV. Thus, we have shown that EPCs treated with IsEMV lose their angiogenic capacity, which is manifested by a decrease in vessel formation *in vitro*. Others have postulated that CKD patients with elevated IS levels may have altered angiogenesis (Hung et al., 2016). In the presence of IsEMV, EPCs reduced the formation of CFUs in a matrix of fibronectin after 21 days of culture. This led us to believe that IsEMV actively interfere in the endothelial repair process.

Furthermore, we observed an increase in NFκB and p53 expression in EPCs treated with IsEMV. It has been described that IS upregulated NFκB and p53 in cells of the proximal convoluted tubule, and NFκB acts by binding to the promoter region of p53 to regulate expression (Shimizu et al., 2010, 2011). This led us to think that IsEMV could act in a similar way. In addition, activation of the NFkB pathway is usually marked by the phosphorylation and degradation of the NFkB inhibitory protein, IkBα, releasing NFkB and allowing its migration to the nucleus (Hinz and Scheidereit, 2014). Accordingly, we observed that increased expression of NFκB is linked with a decrease in the expression of IκBα in EPCs treated with IsEMV.

This effect could be explained by analyzing the miRNAs content of EMV. Our results revealed there are 23 miRNAs in IsEMV and four (miR-181a-5p, miR-4454, miR-150-5p, and hsa-let-7i-5p) were upregulated in IsEMV compared with control EMV. A recent study reported links between miR-181a-5p, miR-4454, and NFκB signaling in cartilage degeneration associated with osteoarthritis (Nakamura et al., 2016). Moreover, 19 miRNAs were downregulated in IsEMV, such as miR-126-3p. This miR-126-3p promotes endothelial proliferation and limits atherosclerosis (Schober et al., 2014); thus, its reduction could explain the limited functional capacity of EPCs (Massy et al., 2017). Moreover, previous studies described that miR-126-3p modulates the expression of NFκB in activated dendritic cells (Agudo et al., 2014) and p53 expression in a murine model of acute lymphoblastic leukemia (Nucera et al., 2016). To date, no study has identified the miRNAs in IsEMV. Our results suggest that miRNAs deregulation probably partly explains the effects of IsEMV on EPCs. Thus, miRNAs, such as miR-4454, miR-181a-5p, and miR-126-3p, could be involved in the increased expression of p53 and NFκB activation by inhibition of IκB. Further studies are required to identify and validate the miRNAs associated with endothelial dysfunction in uremia.

Our study has some limitations related to the instrumentation and protocols used for identifying and obtaining the EMV. The size detection limits of standard flow cytometry are well known, causing smaller MV to be overlooked. The upper size limit of MV detection is likely >1 μm, as a 0.5 μm polystyrene bead reflects an MV around 1 μm (Chandler et al., 2011; Carmona et al., 2017). Consequently, the absolute MV count might be underrepresented. The detection of MV in suspension by flow cytometry has attracted strong clinical and scientific interest, but their detection is difficult because many MV are small (<400 nm), below the limit of resolution of most flow cytometers.

In conclusion, our results show for the first time that IS, a difficult to remove uremic toxin in patients with CKD, is capable of inducing endothelial vesiculation with different characteristics that makes MV potential candidates for studying novel biomarkers being a very useful as diagnostic and prognostic tools for vascular diseases. IsEMV have membrane characteristics, miRNAs, and other molecules that reduce the ability of EPCs to regenerate and participate in the signaling pathways involved in apoptosis and oxidative stress. These specific mechanisms may constitute therapeutic targets in patients with CKD.

## Author contributions

AC, FG, PA, and JC participated in the conception and design of research; AC, FG, PB, and TO performed the experiments, acquired and analyzed data of work; AC, FG, PA, and JC performed interpretation of data; all authors drafted, edited and approved the final version of the manuscript. All authors agree to be accountable for all aspects of the work in ensuring that questions related to the accuracy or integrity of any part of the work are appropriately investigated and resolved.

### Conflict of interest statement

The authors declare that the research was conducted in the absence of any commercial or financial relationships that could be construed as a potential conflict of interest.

## Supplementary material

Data Supplementary Figure 1 Representative images of the formation of vessels in the *in vitro* angiogenesis assay on the semi-natural matrix, Matrigel, 4 h after seeding. **(A)** EPCs treated with EMV or **(B)** IsEMV, **(C)** Vascular endothelial growth factor (50 ng/ml) used as a positive control, and **(D)** Endothelial basal medium (EBM) without fetal bovine serum (FBS) used as a negative control.

Data Supplementary Figure 2 Effect of various concentrations of IS (μg/ml) at different incubation times. Absolute number of MV per microliter in IS-treated and untreated HUVECs. **(A)** 6 h **(B)** 12 h, and **(C)** 24 h of incubation. Results are the mean ± SEM of five independent experiments. ^*^*p* < 0.001 vs. untreated HUVECs; ^#^*p* = 0.002 vs. HUVECs+IS [256].
