# Supplementary figures and images for: Microvesicles Derived from Indoxyl Sulfate Treated Endothelial Cells Induce Endothelial Progenitor Cells Dysfunction
Source: Front Physiol. 2017 Sep 8;8:666. doi: 10.3389/fphys.2017.00666 (PMC5599774; doi:10.3389/fphys.2017.00666)

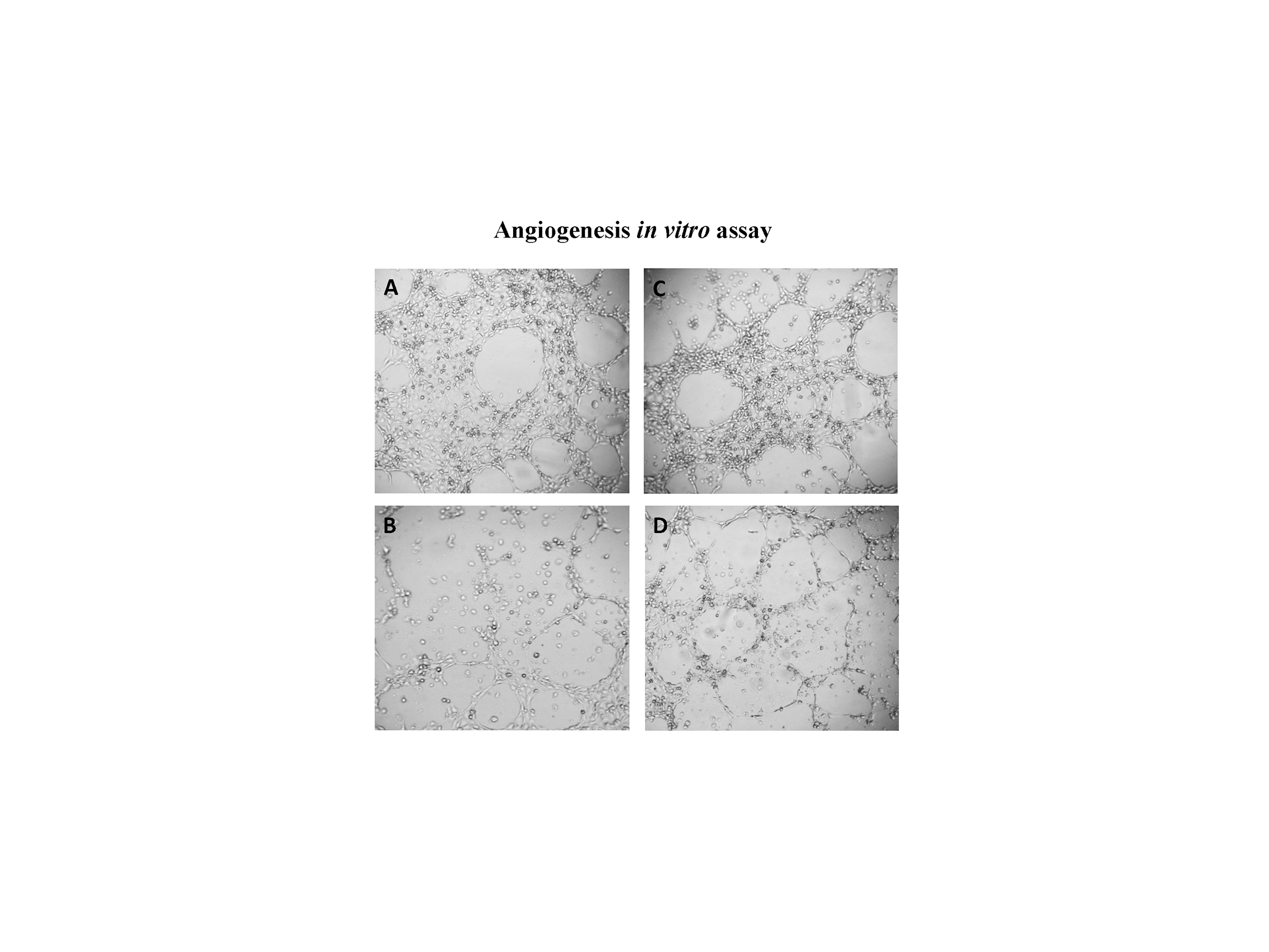

Supplement: Data Supplementary Figure 1 — Representative images of the formation of vessels in the in vitro angiogenesis assay on the semi-natural matrix, Matrigel, 4 h after seeding. (A) EPCs treated with EMV or (B) IsEMV, (C) Vascular endothelial growth factor (50 ng/ml) used as a positive control, and (D) Endothelial basal medium (EBM) without fetal bovine serum (FBS) used as a negative control. [file Image1.TIFF]

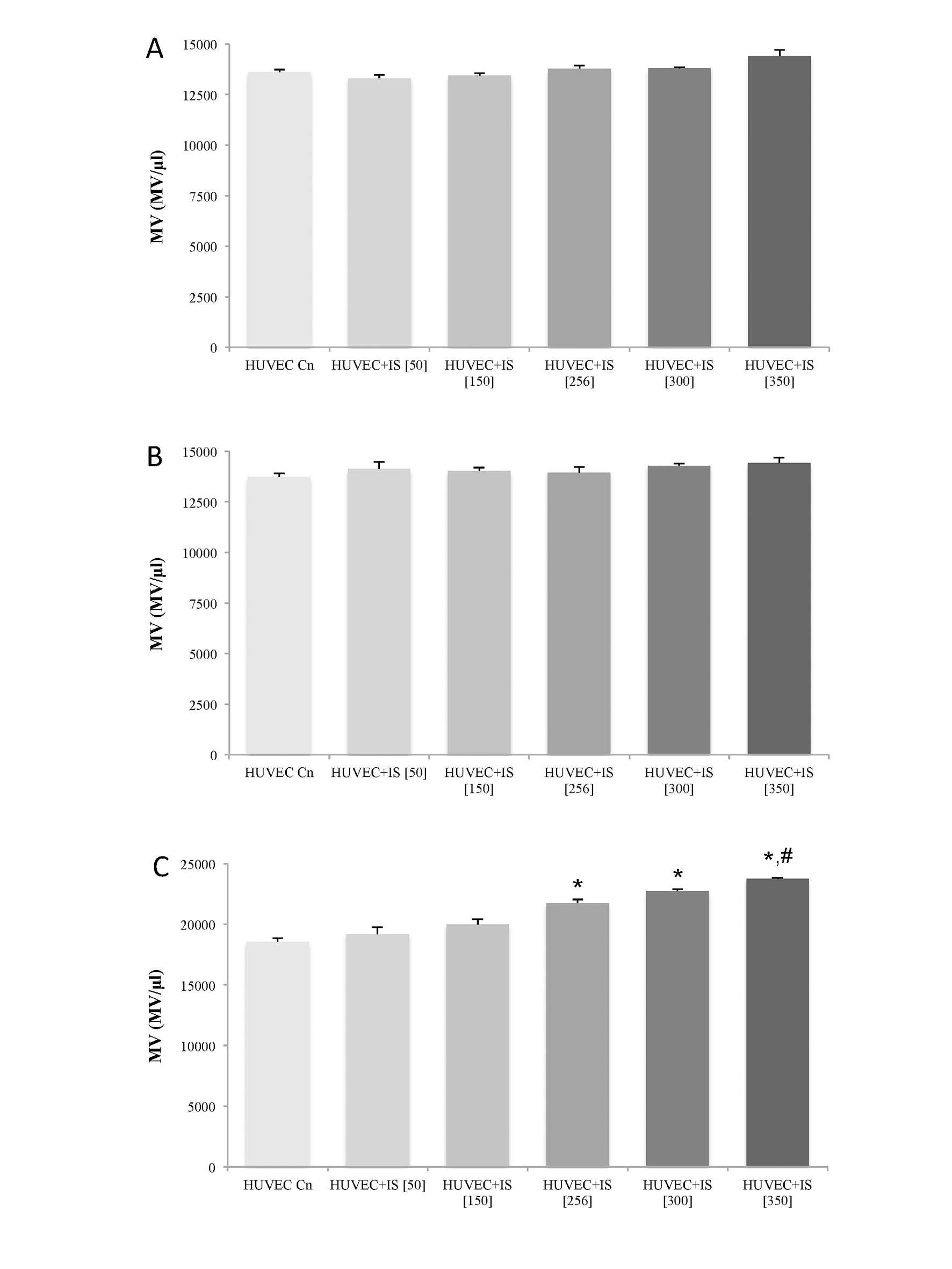

Supplement: Data Supplementary Figure 2 — Effect of various concentrations of IS (μg/ml) at different incubation times. Absolute number of MV per microliter in IS-treated and untreated HUVECs. (A) 6 h (B) 12 h, and (C) 24 h of incubation. Results are the mean ± SEM of five independent experiments. *p < 0.001 vs. untreated HUVECs; #p = 0.002 vs. HUVECs+IS [256]. [file Image2.TIFF]
